# Neglected tropical diseases elimination in Africa: lessons from regional control programmes

**DOI:** 10.1186/s41182-025-00865-8

**Published:** 2025-12-30

**Authors:** Ukam Ebe Oyene, Chukwu Okoronkwo, Honorat Gustave Marie Zoure, Uche V. Amazigo, B. E. B. Nwoke, Moses Nayenda Katabarwa, Thompson Luroni Lakwo, Benjamin Chukwuemeka Nwobi, Nana Kwadwo Biritwum, Joseph Chukwudi Okeibunor, Sunday Isiyaku, Afework Hailemariam Tekle, Nouhou Koncouré Diallo, Latson Douglas Sitima, Elizabeth Osim Elhassan, Boakye A. Boatin

**Affiliations:** 1 Department of Public Health, Faculty of Health Sciences, National Open University, Abuja, Nigeria; 2 National Malaria Elimination Programme, Federal Ministry of Health, Abuja, Nigeria; 3 Former Biostatistics and Mapping Officer, APOC, Ouagadougou, Burkina Faso; 4 Pan African Community Initiative On Education and Health (PACIEH), Enugu, Enugu State Nigeria; 5 Department of Animal and Environmental Biology, Faculty of Biological Sciences, Imo State University, Owerri, Nigeria; 6 Nyange Centre for Health Research and Development (NCHRD), c/o 3457 Thornewood Drive, Atlanta, GA 30340 US; 7 Vector Control Division, Ministry of Health, 15 Bombo Road, P.O. Box 1661, Kampala, Uganda; 8 Foundation for Disease Elimination and Development Legacies, Obafemi-Owode LGA, 46 Maba Road, Off Asheshe Bus-Stop, Lagos-Ibadan Expressway, Owode, Ogun State Nigeria; 9 Former NTDs Programme Manager, Global Health Consultant, Accra, Ghana; 10 Department of Sociology/Anthropology, University of Nigeria, Nsukka, Enugu State Nigeria; 11 Sightsavers, 24 Tennessee Crescent off Panama Street, Maitama, Abuja, Nigeria; 12 Cessy, France; 13 National NTD Preventive Chemotherapy Programme, Ministry of Health, Conakry, Guinea; 14 Ministry of Health, CHSU, P/Bag 65, Lilongwe, Malawi; 15 Freelance Consultant 1a Yusuf Lere Drive, Off Tafawa Balewa Way U/Rimi, Kaduna, Kaduna State Nigeria; 16 20 Trigg Myers Way, Lilburn, GA 30047 USA; 17 3 Kunkun Close, East Legon GPA, 483-9782 Accra, Ghana

**Keywords:** Neglected tropical diseases, Elimination, WHO, Mass drug administration, African region

## Abstract

**Background:**

Africa has made notable progress against Neglected Tropical Diseases (NTDs) using a whole-of-society approach that involved everyone, though sub-Saharan Africa still faces major challenges. Since the Expanded Special Project for the Elimination of Neglected Tropical Diseases (ESPEN) was launched in 2016, over 500 million people have been treated for NTDs like onchocerciasis, lymphatic filariasis, schistosomiasis, and soil-transmitted helminthiases. Nineteen African countries have eliminated at least one NTD, yet 44 out of 52 tracked nations still need preventive chemotherapy for multiple diseases. In 2022, 81 million people received schistosomiasis treatment, but adult coverage remains low due to limited praziquantel access.

This paper documents lessons and success stories from regional initiatives such as the African Programme for Onchocerciasis Control (APOC), the Onchocerciasis Control Programme in West Africa (OCP), and the Guinea Worm Eradication Programme, including success stories from specific countries in implementing NTDs programmes and other health programmes (vaccine-preventable disease programmes, malaria, and some zoonotic disease programmes) for ESPEN and similar partnerships to guide future improvements in NTD elimination efforts.

**Methods:**

Programme reports from the WHO African region on NTDs, and peer-reviewed journals and other published documents were assessed, supplemented by authors’ wide experiences to gain insights into NTDs and communicable disease programme implementation across Africa. The manuscript was structured along the WHO health systems building blocks. Success stories were highlighted, lessons learned documented, and recommendations made. Contents were reviewed several times and independently by a small group. The authors’ approval was secured before journal submission.

**Findings and conclusions:**

Lessons learned, and recommendations made are beneficial to partners and countries in achieving the 2030 NTDs elimination targets in the African region and beyond. They provide pathways to expanding the structure, direction, and scope of programme implementation for greater impact.

## Background

The fight to eliminate NTDs, mainly found in rural settings, urban slums, and low-income countries in sub-Saharan Africa, Asia, and Latin America [1] has dramatically improved with the increased commitment of donors, partners, the private sector, WHO and affected countries and communities, especially in the last forty years. This transformation highlights progress and underscores the need to sustain these efforts. Based on initial successful control efforts through vector control and targeted Mass Drug Administration (MDA), global attention started increasing with the formation of the first international partnership pioneered by Merck in the 1980s for the control of onchocerciasis—one of the NTDs amenable to preventive chemotherapy using ivermectin (Mectizan®)[2]. The successful vector control efforts and targeted MDAs provided the drive for the formation of several coalitions, such as the African Programme for Onchocerciasis Control (APOC) and the Onchocerciasis Elimination Programme for the Americas (OEPA) [3]. Consequently, there has been significant progress recently in controlling NTDs, driven by increased funding from national governments, non-governmental donors, and substantial donations from major pharmaceutical companies [4]. Although progress has been made globally, the most considerable burden remains in sub-Saharan Africa. Climate sensitivity of vector-borne diseases is well recognised [5], yet evidence on climate–NTD interactions remains limited [6]. Conflict, humanitarian crises, and migration further impede elimination [7, 8] while Ebola virus disease (EVD) and coronavirus disease 2019 (COVID-19) disrupted treatments, surveys, and outreach [9, 10]. In response, WHO and partners produced health-emergency guidance and an operational tool [11, 12]. Furthermore, COVID-19 also exposed interdependencies across health systems [13, 14], underscoring the need to strengthen core functions while safeguarding NTD service continuity, accelerating scale-up, and preparing for post-elimination surveillance [15, 16] and climate-resilient action [17].

The Expanded Special Project for the Elimination of Neglected Tropical Diseases (ESPEN) has made significant strides in controlling and eliminating NTDs since its establishment in 2016 by WHO/AFRO, following the closure of APOC. It has coordinated over 500 million people being treated for onchocerciasis, lymphatic filariasis, schistosomiasis, and soil-transmitted helminthes [18]. Several countries have made significant progress in controlling and eliminating various NTDs, especially trachoma, onchocerciasis, and lymphatic filariasis [18]. Nineteen countries in the WHO African Region have successfully eliminated at least one NTD [18].

**Fig. 1:**
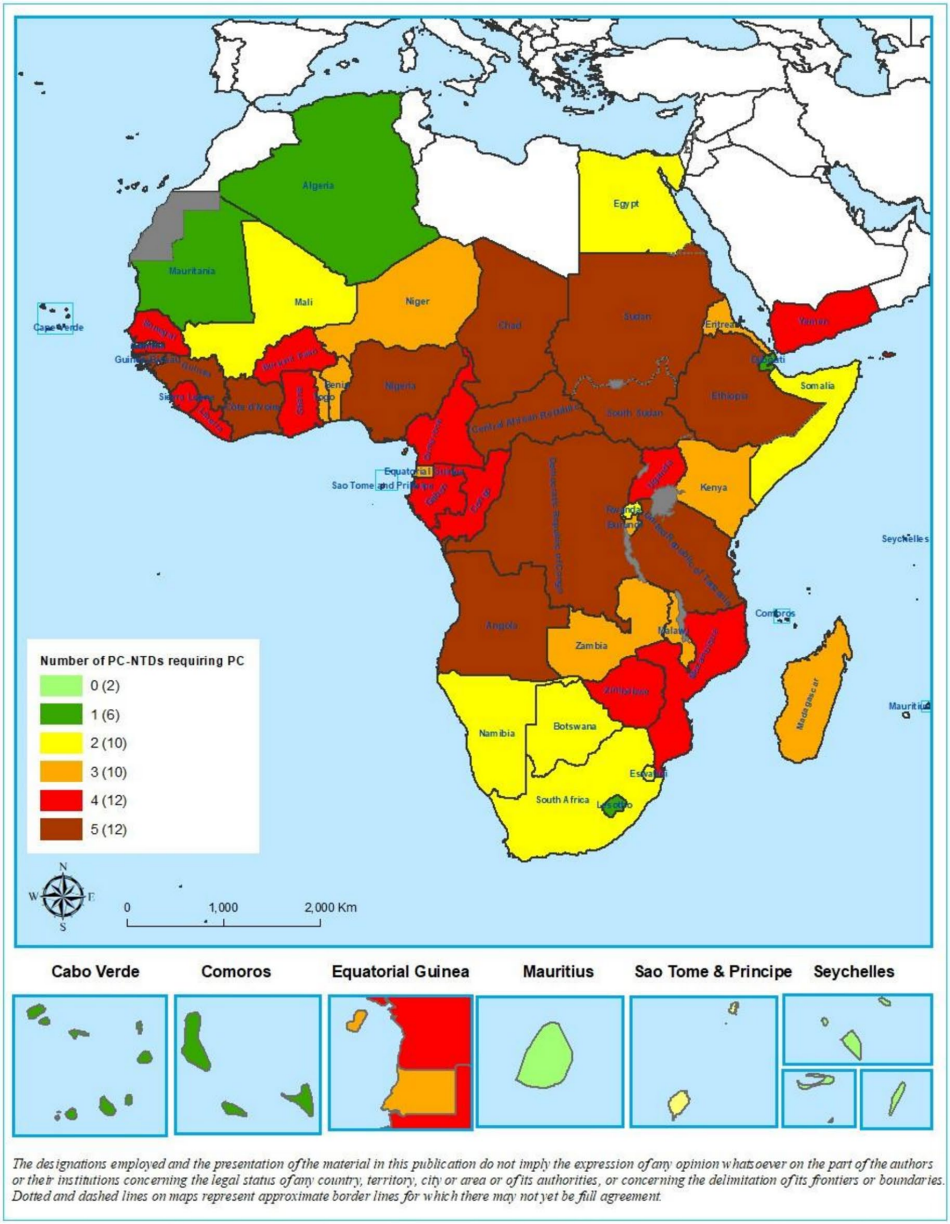
Number of NTDs requiring PC by country (data source: https://espen.afro.who.int. Accessed 30 April 2025). 0 = two (2) countries where no PC is required. 1 = eight (8) countries where one PC intervention is required. 2 = ten (10) countries where two PC interventions are required. 3 = ten (10) countries where three PC interventions are required. 4 = twelve (12) countries where four PC interventions are required 5 = twelve (12) countries where five PC interventions are required

However, among the 52 countries tracked by ESPEN, 44 (85%) still require preventive chemotherapy for 2 or more PC-NTDs (Fig. 1). Although 81 million individuals (school-age children and adults) were reported as having received treatment for schistosomiasis in 2022 in the African region, treatment coverage remains low at 15.2% for adults due to the limited availability of praziquantel for this age group [18].

Critical challenges facing ESPEN include, but are not limited to, significant programme underfunding, weak country-led coordination among partners, inadequate cross-sectoral engagement to address key NTDs’ determinants, and health system challenges such as insufficient disease surveillance capacity, a shortage of health workers, and limited integration in many countries. [19]. A scoping review identified varying levels of gaps in technical, strategic, and service delivery across countries in the WHO/AFRO region [20]. These challenges are familiar and have plagued other control or elimination programmes, including OCP and APOC [21–23]. ESPEN management and other key stakeholders/partnerships will benefit from lessons learned from successful previous and present regional programmes, including well-managed epidemics, to address these challenges and achieve the objectives.

Specific objectives are:

- Examine NTD programme implementation across the WHO African Region, including the 11 countries previously under the Onchocerciasis Control Programme (OCP), the 20 former APOC countries, and 52 countries currently engaged through ESPEN.
- Document notable success stories in public health programme delivery and implementation.
- Provide actionable recommendations to strengthen NTD programming and advance elimination efforts within the African region.

## Methods

**Fig. 2:**
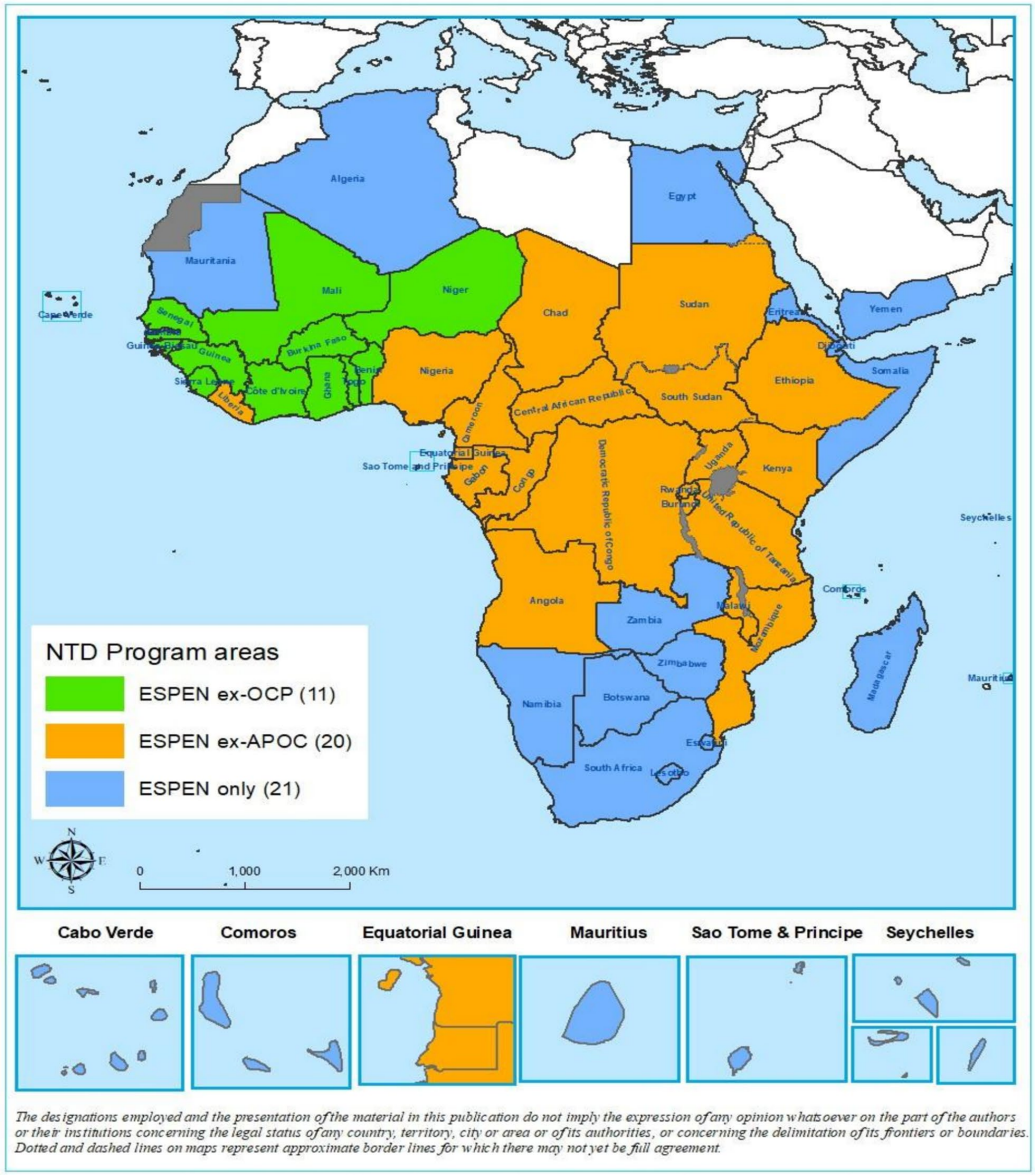
Map showing NTD programme areas (map template adapted from WHO/AFRO/UCN)

This study covers the WHO African region, encompassing 11 countries previously part of OCP, 20 former APOC nations, and 52 countries currently participating with ESPEN (see Fig. 2). Although the analysis centres are in the African region, the results are broadly relevant. We developed a structured writing framework to ensure comprehensive documentation of lessons learned, developed collaboratively by a multidisciplinary team, during the planning stage. We held virtual meetings, including email communications, to guarantee that the manuscript addressed the essential requirements of NTDs programmes, and we generated evidence from expert opinions, leveraging the collective experiences of all co-authors on PCT-NTDs and other infectious diseases programming.

**Fig. 3:**
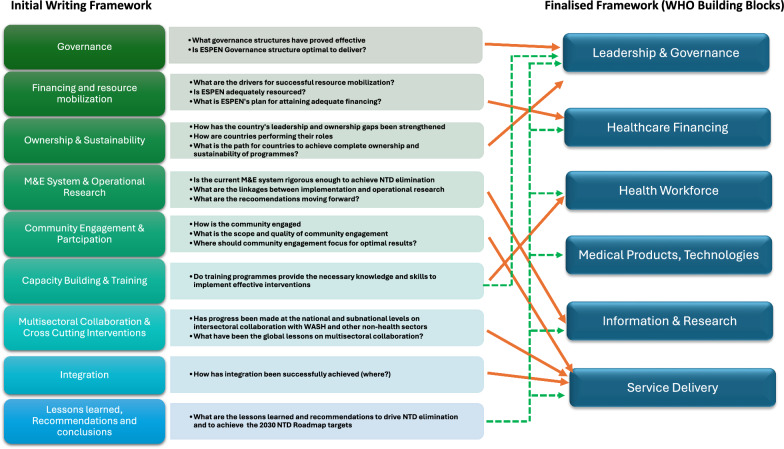
Writing framework

We searched NTD partners’ websites and the ESPEN portal for Master Plans, operational plans, reports, and coverage data pertaining to NTDs in various countries. Additionally, we sourced programmatic information from the WHO Institutional Repository for Information Sharing (IRIS) database and the WHO quarterly bulletin. We also conducted a literature search, including case studies and peer-reviewed articles, identified through PubMed, Google Scholar, and related databases using targeted keywords from questions in the writing framework (Fig. 3).

We utilised geographic terms to focus our search results on specific countries and regions. Publications in English and French were included, and the year of publishing had no restrictions. Articles from the searches that provide data on NTDs and other infectious diseases, such as smallpox and malaria, together with their achievements, gaps, challenges, best practices, innovations, and lessons learned, were major criteria for the selection of articles. Listing of articles in the reference manager helped with prioritisation, we cited 134 articles that addressed the needs of this paper. The writing format was derived from WHO’s systems building components, and we included determinants; Sacks et al. [24]. Before submitting the work to the journal, the co-authors conducted internal and editorial reviews and approved the final draft.

## Lessons, evidence, and applications for NTDs

### Leadership/governance

#### Governance

Providing oversight and coordinating disease control programmes at regional and global levels could benefit from flexible and pragmatic governance structures aimed at the interests of country NTDs programmes [25]. ESPEN was established to enhance country ownership and capacity, prioritise other preventive chemotherapy (PC) diseases equally, and provide funding organisations with greater flexibility in their donations [26, 27]. Its lean staff is embedded within the WHO/AFRO administrative structure, with the Steering Committee reporting to the Regional Director through the Director of Programme Management while maintaining links with the Regional Programme Advisory Group (RPAG).

APOC and its predecessor, the OCP, oversaw the control of onchocerciasis in Africa from 1975 for over 40 years [28]. They had similar governance structures—a comparatively lean staff at headquarters, with some technical support staff in identified high-burden, low-capacity countries, embedded in the country offices of WHO. APOC had a Technical Consultative Committee (TCC), Joint Action Forum (JAF), and the Committee of Sponsoring Agencies (CSA) [28]. OCP also had the Joint Programme Committee (JPC), the equivalent of APOC’s JAF, the CSA and the Expert Advisory Committee (EAC), the equivalent of APOC’s TCC, with its subsidiary, the Ecological Group.

Under APOC, there was depth and comprehensiveness at APOC’s JAF meetings, along with the attendant sense of belonging, buy-in from all the countries, and desk officers supporting some countries’ NTDs programmes.

#### Leadership

Country ownership and strong country leadership are critical for meeting the 2030 NTDs elimination targets, given that countries are the drivers and beneficiaries of any progress made [1]. In demonstrating ownership, governments typically house most NTDs programmes within the Ministry of Health, which benefits from office space, staffing, transportation, communication, and other relevant oversight and technical guidance. Additionally, some endemic countries have integrated some NTDs into well-funded programmes and incorporated them into national surveillance, district routine activities, and drug delivery systems. In several countries, governments support the development and implementation of District Implementation Plans (DIPs). A few countries made considerable efforts in resource mobilisation, and their leaders have led fundamental transformations in health on the continent [29]. However, though NTDs are usually reflected in many countries’ health sector’s overall budgets, delays or lack of release of these funds for activities have been documented [30]. In most countries, healthcare interventions rely on donor funds, and budgetary allocation to health services is typically below 15 per cent, despite the Abuja Declaration, and this represents a significant underinvestment in healthcare, particularly for NTDs [31].

Conversely, delayed or unreleased budget lines, dependence on external funds, and shortfalls against Abuja health-financing commitments have repeatedly stalled execution. The recent suspension of some donor-supported programmes highlights the fragility of parallel delivery channels and the necessity of institutionalising NTD functions within government systems to avoid reversals, and the need for systems thinking [32, 33].

Under ESPEN, the African Leaders Malaria Alliance (ALMA) scorecard, promoted by the Uniting to Combat NTDs group and the African Union, has been enhanced, and other checklists have been utilised to measure country contributions and progress transparently. The strong advocacy from WHO galvanised global political commitment, facilitating decentralised regional and national programme leadership and management, which led to a successful smallpox eradication programme [34]. APOC adopted practical and impactful approaches to improving country ownership by tracking progress in financial and in-kind contributions at national, district, and community levels and for supporting Community Drug Distributors (CDDs) [35]. Additionally, to strengthen domestic resource mobilisation, APOC hired experts in resource mobilisation to train country teams. Participating countries shared all these experiences during annual review meetings.

Levine [36] highlights that strong advocacy and political will were crucial in eradicating Guinea worm disease. Prominent leaders like the late US President Jimmy Carter, Nigeria’s former Military Head of State, General Yakubu Gowon, Mali’s President Amadou Touré and other African leaders repeatedly visited countries to ensure fulfilment of commitments, provision of the necessary resources and stressed the importance of global coordination through regular interagency and national eradication meetings that facilitated partner and donor collaboration.

Leadership played a significant role in managing the EVD outbreak in Nigeria, Senegal, and Mali. Otu et al. [37] reported that in Nigeria, funds were allocated and distributed by national and subnational governments, where guidance from the Ministries of Health and the WHO facilitated procurement of supplies, establishment of isolation facilities with existing polio equipment and surveillance systems being used for contact tracing and mapping transmission chains.

### Health care financing

#### Financing and resource mobilisation

An efficient and transparent fund management system, along with sustainable resource mobilisation, is paramount to eliminating NTDs [38]. The Kigali Declaration and its unanimous endorsement are crucial to achieving the goal of eliminating NTDs. Under the current arrangement, partners and donors can support countries directly, while ESPEN attempts to fill the gaps. In 2023, ESPEN had just a little above 15 million USD for such gap-filling [19]. Recently, the Uniting to Combat NTDs group collaborated with the Federal Ministry of Health, civil society organisations, and development partners in Nigeria to conduct high-level advocacy at national and subnational levels, targeting increased domestic financing of NTDs. The launch of the Reaching the Last Mile Fund (RLMF) in 2018, working with ESPEN in the WHO Regional Office for Africa and providing funding to 24 countries in 2024, with plans to fund 39 countries in 2026, certainly invigorated the elimination of onchocerciasis and lymphatic filariasis in the African Region [39]. The report of the APOC evaluation indicated that financial assistance primarily came from Trust Funds, AFRO, the Mectizan Donation Program, and the Bill and Melinda Gates Foundation [26]. Overall, the partnership with key stakeholders in its resource mobilisation drive was the bedrock of APOC’s success.

Sustained focus and continuity in resource mobilisation propelled the APOC to achieve onchocerciasis control [40, 41], a reduction in skin and eye lesions, and a significant decrease in the skin microfilaria load of *Onchocerca volvulus* [41]*.* Evidence in 2009 of large-scale treatment with ivermectin stopping further infections, especially in areas with high percentages of treatment and population coverage, led to the shift from control to elimination of onchocerciasis transmission [41]. Undoubtedly, countries that have been verified or validated in the last 5 years for the elimination of any of the PC-NTDs, viz. Benin, Gambia, Ghana, Malawi, Mali, and Togo, for trachoma; Togo and Malawi for LF; and Niger for onchocerciasis, made strong resource mobilisation efforts to achieve these feats and these countries should document such lessons for easy reference, including those from the APOC’s robust platform for the resource mobilisation drive.

The Global Guinea worm eradication programme is successful and on the verge of eradication, thanks to sound technical support from the WHO, CDC, and UNICEF; strong and continuous political advocacy; and sustained financial support from governments and NGOs. Only six countries remain to be certified as having eliminated Guinea worm disease: Angola, Chad, Ethiopia, Mali, South Sudan, and Sudan [42–44]. The involvement of The Carter Centre in dracunculiasis eradication played a crucial role in the global campaign, as well as the advocacy role of the late President Jimmy Carter, which secured donor funding. Coupled with the personal commitments of former and current Heads of State in some countries, the late President Jimmy Carter travelled to Africa for campaigns and brokered the ‘guinea worm ceasefire’ of 1996, facilitating active case searches in Sudan’s conflict zones [45]. Similar initiatives could help address PCT-NTD elimination in other conflict zones in Africa [46].

Eliminating neglected tropical diseases will require a long-term commitment from national and global financing partners [46]. Funding must address systemic gaps and should be allocated as part of the government’s communicable diseases programmes—malaria, HIV/AIDs, hepatitis, NTDs and TB [47]. Advocacy for government and private sector funding is necessary to support holistic programmatic interventions and the Programme for Research and Training for Neglected Tropical Diseases**.** Africa has much to learn from the China Schistosomiasis Elimination Programme [48]. The China schistosomiasis experience reinforces that domestic fiscal commitment and integration with major financing vehicles, such as the Global Fund to fight AIDS, TB and Malaria (GFATM), are essential to prevent resurgence after external support ends [49].

### Service delivery

#### MDA and beyond

ESPEN-supported programmes have delivered hundreds of millions of PC treatments, leveraging community-directed models (originating under APOC), school-based delivery, and facility-based approaches, often in combination to optimise coverage [41]. There are notable gaps in Mass Drug Administration (MDA) coverage for certain PCT NTDs, in certain countries and specific areas achieving below the recommended threshold for soil-transmitted helminthiases and schistosomiasis [50], as well as for onchocerciasis and lymphatic filariasis [39]. The rationale for highlighting *Loa loa* co-endemic Central African settings is clear. They present the ideal “no one-size-fits-all” constraint, where the risk of severe adverse events demands tailored strategies, including RAPLOA, LoaScope, while addressing waning enthusiasm by populations to continue MDA [51–53], test-and-treat, and targeted deployment to progress without harm [53–55]. Examples of successful Triple Drug Therapy pilot in Africa and deployment to scale in Pacific nations are well-documented [55, 56]. Logistics remain a determining factor, where investments by Guinea worm, APOC, and NGDOs in transport and field operations are directly translatable to strengthening last-mile supervision and drug accountability today.

#### Disease management and disability inclusion (DMDi)

Disease management and disability inclusion (DMDi) mark a strategic shift in neglected tropical disease (NTD) programming from a focus on transmission control toward a comprehensive, person-centred approach that integrates early diagnosis, case management, morbidity management and disability prevention (MMDP), rehabilitation, and social inclusion within national primary health care (PHC) systems [1, 57–59]. Central to these efforts is the integration of Skin-NTD platforms, supported by innovations such as the WHO Skin NTDs mobile app, which enhances frontline diagnostic accuracy and decision-making in low-resource settings [60] and the establishment of the Skin NTDs Laboratory Network and ESPEN Laboratory, which strengthen diagnostic quality, training, and surveillance across the African region [61]. While these tools represent significant gains, challenges persist, including underinvestment relative to preventive chemotherapy interventions, limited uptake and supervision of digital tools, gaps in laboratory infrastructure, supply-chain constraints, and service disruptions in fragile contexts [62]. Successful implementation examples from Malawi, Tanzania, and Nigeria demonstrate the potential of integrated community-led and digital DMDi models, particularly for lymphatic filariasis, leprosy, Buruli ulcer, female genital schistosomiasis, and trachomatous trichiasis, but highlight ongoing barriers such as transport costs, consumable shortages, and stigma [63–65]. To close these gaps and [62] achieve universal health coverage and elimination targets, African countries must scale and finance DMDi services within national benefit packages, expand district-level surgical and rehabilitation capacity, improve diagnostic networks, integrate disability indicators into health information systems, and co-design services with affected communities to ensure equity, quality, and sustainability [66].

#### Multisectoral action and One Health

Because NTD determinants [59] lie in WASH, housing, livelihoods, animal reservoirs, and ecosystems, durable gains require whole-of-government action [67, 68]. The One Health approach emphasises the strong link between human, animal, and environmental health, as well as their impact on the ecosystem and the ESPEN countries in the WHO African region’s determination to involve stakeholders outside the health sector in the planning process [64]. Furthermore, Guinea worm–WASH collaboration in Nigeria and Uganda exemplifies negotiated alignment between health and water sectors that delivered infrastructure, community ownership, and functional maintenance [69, 70]. Low utilisation of water points or latrines highlights the imperative for behaviour-centred design and social and behaviour change communication to convert infrastructures into tangible health [71]. Climate- and environment-linked re-emergence, zoonotic reservoirs, and pastoralist contexts argue for proactive One Health surveillance and environmental health within post-elimination monitoring [72–74] and Health-in-All-Policies approaches [75], and lessons from rinderpest eradication further justify formal cross-sector mechanisms [76].

#### Cross-border collaboration and mobile populations

Cross-border collaboration is crucial because transmission zones ignore borders; elimination demands synchronised action [77]. OCP/APOC’s cross-border interventions replicated under ESPEN are included for their proven ability to align testing, MDA calendars, vector evaluation, and data exchange across contiguous settings [78, 79]. The Global Network for Onchocerciasis Elimination (GONE) is commended for organising webinars between bordering countries to exchange data on neighbouring districts and the challenges to be addressed regarding the cross-border action of NTDs elimination [80]. The Galabat–Metema experience in Ethiopia, Sudan border, illustrates both the success of coordinated stopping and the necessity of vigilant post-intervention surveillance to detect resurgence [81]. Samoa–American Samoa coordination around IDA demonstrates the same principle beyond Africa [56]. Seasonal labour migration, displacement, and conflict complicate coverage and surveillance [82, 83].

#### Community engagement and participation (lessons, evidence, applications for NTDs)

A key lesson from the implementation of community engagement in onchocerciasis control is its relevance as a tool and a crucial contributor to service delivery and utilisation for eliminating NTDs in Africa. This underscores the urgency and importance of community engagement in the fight against NTDs; therefore, elimination programmes should prioritise adaptability in local contexts and strengthen community institutions to foster a shared understanding [84, 85], including innovative, people-centred designs [86].

Under the smallpox eradication programme, support was mobilised from civil society organisations, local leadership, and communities to develop strategies adapted to local conditions, addressing “last mile” eradication challenges [87]. With APOC, community engagement was the fulcrum and pillar of the successful onchocerciasis control in Africa, a phenomenon well-documented [88–90] which yielded positive results, demonstrating impact, inclusivity, and cost-effectiveness. They fostered community empowerment, ownership, and buy-in, thereby ensuring onchocerciasis control and elimination in some foci and increasing coverage in a large part of Africa [40, 41]. APOC and its NGDO partners were able to reach many neglected, hard-to-reach communities that had limited access to orthodox health services. The structure also served the successful implementation of an entomological evaluation of onchocerciasis control, in identifying, prospecting vector breeding sites, capture points to set and protect black fly traps, and conducting human landing collections of female black flies [91]. Community-centred approaches are helpful to scale up local vector control initiatives using molluscicides and modifying the environment in areas with high schistosomiasis transmission.

Weaknesses in deploying the APOC strategy included low cohesion within specific communities [30, 35], mainly due to inadequate skills of frontline health personnel to facilitate proper engagement, poor planning, and a need for an enabling policy environment for community and citizen participation in planning and executing health interventions. However, lessons can be learned from South Africa’s policy reforms, which have institutionalised community participation in health and development [92, 93]. In Vanuatu, Southwestern Pacific, the malaria elimination programme with sustained community support motivated people to adhere to elimination interventions [94].

In terms of application for NTDs, it is important to devise appropriate community engagement strategies in areas that have undergone more than 15 years of MDA and treatment fatigue has set in, as well as in NTDs hypo-endemic areas with no prior MDA experience. Polio SIA practices, mapping nomadic routes, engaging clan leadership, deploying trusted mobilisers and volunteer vaccinators, offer concrete strategies for NTDs in mobile populations [74, 95–98].

#### Integration and primary health care

The WHO NTD Roadmap calls for mainstreaming NTDs into national systems and integrating across programmes [1]. ESPEN’s master-plan reviews now interrogate the depth of integration across PC and case-management NTDs [99]. Rwanda’s scorecard-enabled integration of malaria, schistosomiasis, and STH into PHC, and Niger’s integrated malaria–NTD model, are included because they demonstrate practical PHC institutionalisation pathways [100].

APOC’s co-implementation experience and subsequent TDR-guided operational research showed how interventions can be layered efficiently when aligned with the same populations [101–103]. Integration with emergency management further protects continuity of essential services during shocks, echoing WHO guidance that equips health workers for disaster response. Coordinated efforts between NTDs, communicable disease programmes, broader health services and emergency management are vital for a sustainable health system. Maat et al. [104] discussed initiatives in Sierra Leone, Madagascar, Uganda, and Ethiopia that integrated responses to diseases and emergencies.

### Information and research

#### Mapping

WHO/ESPEN and partners have invested heavily in mapping, building on REMO and geostatistical methods to fill baseline gaps, delineate transmission zones, and prioritise surveillance [19, 105]. The current gap in mapping the PC-NTDs in the WHO African Region lies in Onchocerciasis Elimination Mapping (OEM), and resources are required to address the mapping gaps with focus shifted to the delineation of transmission zones, including hypo-endemic areas. The principles that guided REMO remained fundamental in delineating transmission zones [106]. The Rapid Assessment Procedure for Loiasis (RAPLOA) method, which involves surveying selected villages through spatial sampling, is still relevant for mapping *Loa loa* high-risk areas [107].

#### Surveillance, monitoring and evaluation

Surveillance weaknesses lead to underdiagnosis and under-reporting, particularly for non-helminth NTDs [18]. ESPEN has supported extensive TAS/iTAS, OEM, and impact assessments, while USAID’s ADAPT provides a structured method to diagnose survey failure and design remedial actions [19, 39, 108, 109]. The convergence of RLMF financing with ESPEN technical support aims to close high-priority assessment gaps for onchocerciasis and LF. Historical precedents from smallpox and Guinea worm, measurable objectives, feedback loops, village-level surveillance, active case search, and intercontinental data sharing remain highly relevant [87, 109, 110]. APOC’s community self-monitoring is a ready-made mechanism for social accountability and continuous quality improvement at the community level that should be revitalised [111].

#### Data analytics and digital platforms

ESPEN built on data management capacity provided by OCP and APOC by leveraging the latest technological advances to add the regional dimension through its portal. The ESPEN portal (https://espen.afro.who.int/) is a digital platform that brings together a set of resources (reports, guides, maps, graphs, interactive dashboards), data collection tools (ESPEN Collect) and communication channels (JAP Upload tool) between stakeholders in the fight against NTDs (Ministries of Health, WHO, Implementing Partners and Donors), and data analysis tools, including generative artificial intelligence. By aggregating data on disease epidemiology, treatment coverage, and disease-specific monitoring and evaluation frameworks on user-friendly dashboards, the ESPEN portal enables stakeholders to better identify communities that need to assess the epidemiological situation, with a view to stopping drug distribution or adjusting the disease control strategy. According to the WHO/ESPEN 2023 Annual Report [35], the number of new users on ESPEN’s portal increased by 93% between January 2022 and July 2023, indicating a notable increase in engagement [112, 113]. Whilst these are welcome developments, there is a critical need to strengthen data analytics capacities in countries for NTDs programmes to track performance status, among other parameters [14]. Additionally, there is a need for further optimisation of these tools and practices.

#### Operations and implementation research

ESPEN encourages harnessing results from operations and implementation research projects to improve implementation efforts. Operations and implementation research were the bedrock of OCP and APOC. Remme enumerated the contributions of WHO/TDR to advancing research in onchocerciasis control [114]. Research conducted by the OCP in 1974 and the APOC Programme from 1998 to 2015 are quite relevant to the current goal of NTDs Elimination [61].

The OCP collaborated with Erasmus University Rotterdam in developing the ONCHOSIM model. An independent review of the OCP led to the WHO/TDR research that established APOC and facilitated the partnership with Merck & Co. Most national onchocerciasis control programmes began in the late 1940s and early 1950s. They pioneered small-scale programmes, often supported by colonial governments or the World Health Organization (WHO). They focused on treating individual patients with diethylcarbamazine citrate (Hetrazan) and suramin, both of which triggered severe adverse reactions and sometimes death [115]. The OCP’s independent review led to the establishment of the Onchocerciasis Chemotherapy project by the Joint Programme Committee and the WHO TDR Filariasis group, resulting in the discovery of ivermectin for treating onchocerciasis [116]. The pioneers also employed vector control methods, including the use of Dichlorodiphenyltrichloroethane (DDT), ground larviciding, and bush-clearing [116, 117].

With the availability of donated ivermectin in 1987, community-based and mobile treatment methods were introduced. However, consistently attaining adequate treatment coverage has always been challenging for programmes. The TDR launched a multi-country study on community-directed treatment with ivermectin (CDTI), which proved more successful in attaining an adequate treatment coverage of at least 80% of the total affected population [3, 118]. It is still one of the most successful methods of mass chemotherapy today, and without adequate treatment coverage, the elimination of neglected tropical diseases (NTDs) will remain an unattainable goal.

While OCP relied mainly on vector control, it was through its support for testing that ivermectin was found to be safe and effective for human use against onchocerciasis [119]. After 37 years, ivermectin is still the medicine of choice for treating onchocerciasis. APOC's support for the CDTI programme in Cameroon encouraged the study to investigate whether doxycycline treatment can be delivered using the CDTI approach. The study demonstrated that empowered communities could effectively deliver doxycycline for 6 weeks to the eligible population through community-selected distributors, achieving good treatment coverage [120].

OCP was at the forefront of studying the effectiveness of different insecticide compounds against *Simulium* vectors, as well as their environmental impact. These included Dichlorodiphenyltrichloroethane (DDT), an organochlorine and synthetic insecticide; Temephos (Abate), an organophosphate larvicide; and *Bacillus thuringiensis*, a biological larvicide. Although DDT was later banned, Temephos is still in use today and is occasionally applied to complement MDA, eliminating onchocerciasis and controlling mosquitoes that transmit malaria and lymphatic filariasis [117]. OCP-assisted studies also played a significant role in understanding the morphological and molecular identification of *Simulium* and non-*Simulium* vectors. Because of this knowledge, onchocerciasis elimination programmes can collect and identify the vectors, using them to evaluate the progress of the elimination programmes. OCP was also instrumental in developing and using the polymerised chain reaction (PCR) technique for analysing collected *Simulium* adult female vectors [121]. It is based on deoxyribonucleic acid (DNA) and is strongly recommended by the WHO verification guidelines for evaluating the progress of interruption and eliminating transmission of *O. volvulus*.

Previous regional programmes have tried to mainstream research and innovation into countries’ NTD programming. Each country has its unique implementation challenges, and any operational research should be geared towards producing local solutions that have global applications.

### Health workforce

The NTDs programme is strengthening capacity across multiple levels with support from ESPEN, partners, and participating countries. Despite ongoing initiatives, the programme experiences substantial attrition due to staff transfers, retirements, and reduced motivation. A primary challenge lies in developing, retaining, and consistently improving capacity until full integration or disease elimination is reached, after which the programme transitions to surveillance activities. In 2023, ESPEN supported capacity-building for 317 national-level personnel spanning 31 countries, covering areas such as NTD data management, entomological and epidemiological surveillance, and the preparation of disease elimination dossiers.

Gender inclusion is integral to reaching NTD elimination goals in Africa [122]. To advance this priority, WHO/AFRO, guided by ESPEN and in partnership with key stakeholders, has extended efforts in leadership development and gender equity through the launch of the Mwele Malecela Mentorship (MMM) Programme for women in NTDs. This programme seeks to empower women as influential leaders at the national, regional, and global levels [19].

OCP and APOC have made distinct contributions towards capacity building in endemic countries. Between 2009 and 2012, APOC offered long-term fellowships, enhancing countries’ competencies in controlling NTDs and supporting health ministries in advancing disease elimination within Africa. APOC’s long-term fellowships created cadres who continue to anchor national programmes. The selection of these initiatives reflects a simple logic that sustained elimination requires institutional memory and interdisciplinary teams in epidemiology, entomology, anthropology, implementation science, and data systems, further supported by supervision, incentives, and logistics that keep community networks viable [123, 124].

The WHO and partners are providing free online courses for programme managers and health professionals, and the integration of training and skill sharing within the disease clusters is increasing, but requires further expansion to benefit the broader health sector. The institutionalisation of NTD training is slowly gaining pace at the country level in the WHO African region, with examples from Rwanda and Niger [100].

The APOC partnership delivered training to community-directed distributors and health workers [26], a process that has been expanded and sustained through countries, ESPEN’s and partners’ investments. As noted by Amazigo et al. [23], these trained personnel function at the frontline in addressing neglected tropical diseases. Their capacity now plays a vital role in controlling NTDs, preventing malaria, and implementing other essential health interventions. Continued investment is necessary for capacity building, supportive supervision, incentive structures, motivation, and network strengthening to ensure sustainable operations at the community level [92].

### Medical products, technologies

The availability and coordinated delivery of donated medicines for preventive chemotherapy-targeted neglected tropical diseases (PC-NTDs) is one of the greatest achievements in global public health. It reflects unprecedented public–private partnerships and large-scale pharmaceutical philanthropy. In 2024, 1.8 billion tablets of NTD medical products were supplied to countries, with 910 million of these provided through WHO facilitation [125]. Globally, 29.5 billion tablets have been delivered over the last 14 years, with 16.6 billion coordinated by the WHO [125] These achievements demonstrate strong coordination, equitable resource allocation, and sustained political commitment.

At the global level, programmes such as the International Trachoma Initiative (ITI), the Global Polio Eradication Initiative (GPEI), and UNICEF’s Supply Division exemplify how sustained financing, pooled procurement, and dedicated logistics infrastructure enable uninterrupted service delivery. ITI’s Zithromax® donation programme, supported by Pfizer, has delivered over one billion doses to more than 30 countries while maintaining on-time delivery rates above 95% [126]. GPEI and UNICEF achieved synchronised campaigns across continents through pooled procurement, advanced market commitments, and real-time data tracking to enhance decision-making [127]. These programmes illustrate how logistics autonomy, sustained financing, and strong partner coordination translate into operational resilience, which form key lessons for NTD programmes advancing toward 2030 targets.

In contrast, the WHO NTD medicine donation programme, although comparable in scale, operates with fewer dedicated resources for warehousing, in-country transport, and last-mile monitoring. Global programmes targeting five preventive chemotherapy neglected tropical diseases (PC-NTDs) have scaled up rapidly in recent decades due, in large part, to the generous drug donations from six pharmaceutical companies—Eisai; Johnson & Johnson (J&J); GlaxoSmithKline (GSK); Merck & Co., Inc., Kenilworth, New Jersey, United States of America (MSD); Merck KgaA; and Pfizer. Today, the scale of the PC-NTD drug donation programmes is staggering. 15 billion tablets have been produced and delivered to those in need, supported by complex supply chains designed to manage operations at this scale.

At regional and national levels, ESPEN’s coordination has supported countries with regulatory navigation, forecasting, and applications for medicine donations. Since its establishment, ESPEN has facilitated over 500 million treatments and managed 4.1 billion tablets, contributing significantly to the control and elimination of onchocerciasis, lymphatic filariasis, schistosomiasis, and soil-transmitted helminthiases [19]. In 2023, ESPEN facilitated more than half a billion tablets for MDA interventions and supported applications for nearly 260 million tablets planned for 2024 [19]. Limitations in the donation scope, notably for adult praziquantel, necessitate countries to begin domestic procurement. However, gains remain vulnerable due to persistent supply chain disruptions exacerbated by the COVID-19 pandemic [128], withdrawal of funding for MDA interventions, and import restrictions in countries with limited domestic manufacturing capacity. This underscores both the strength of the current system and its fragility without long-term sustainable financing and national ownership [129, 130].

However, national regulations on the importation of albendazole, mebendazole, and tetracycline due to domestic manufacturing policies pose new challenges, where local production cannot match the quality or volume requirements.

At the community level, equitable access hinges on last-mile logistics, accountability, and real-time data visibility. Digital logistics management information systems (LMIS), mobile technologies, and GIS mapping are required to improve inventory tracking and forecasting accuracy [131]. There are opportunities for countries to pilot and scale up artificial intelligence-driven forecasting tools that link epidemiological and demographic trends to distribution needs, reducing wastage and preventing stockouts [132].

These technological advances accelerate elimination progress only when local institutions are empowered to manage, adapt, and sustain them. However, the role of endemic rural communities, central to implementation and long-term ownership, remains insufficiently defined and requires clearer articulation.

### Adapting learnings to diverse settings

**Fig. 4:**
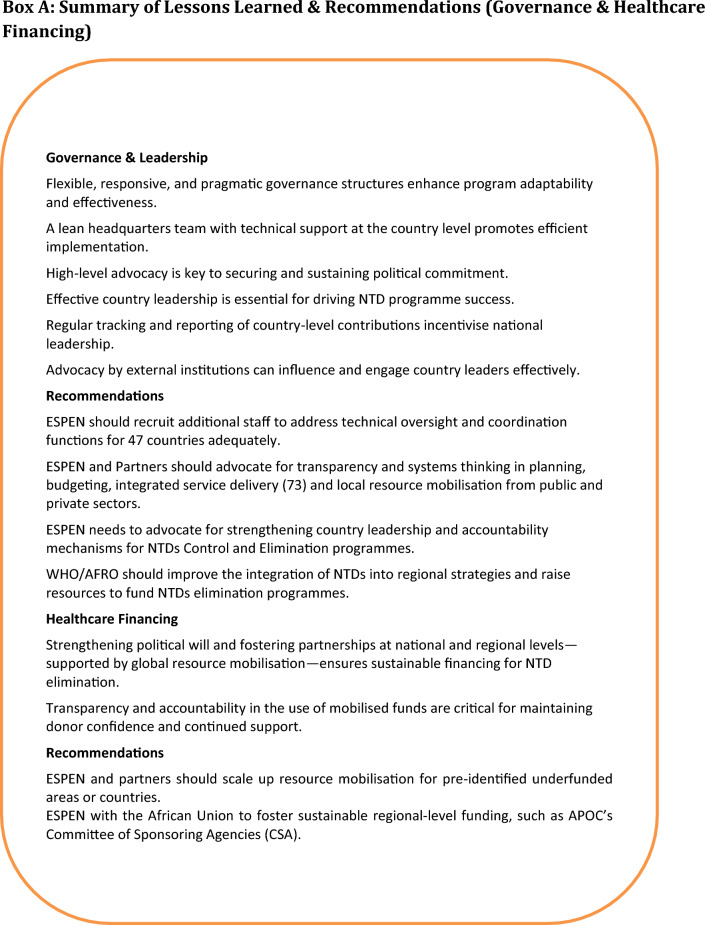

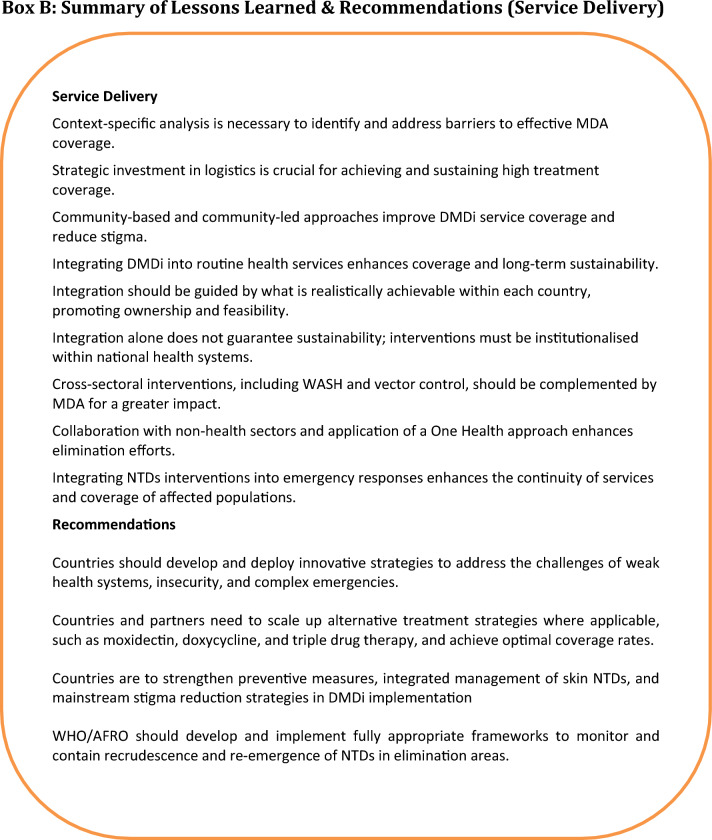

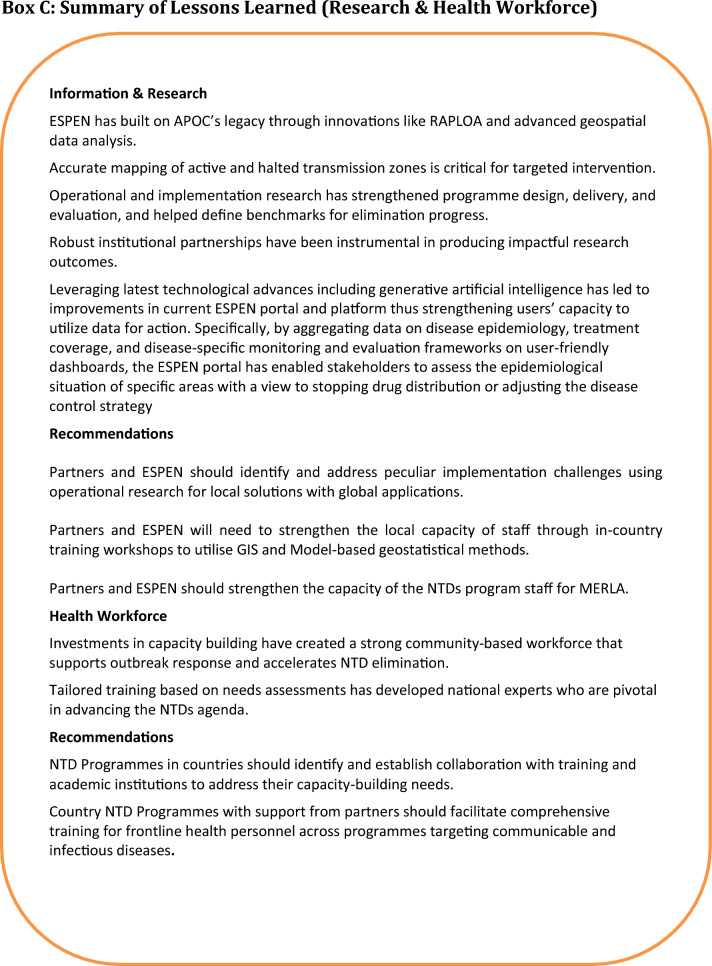

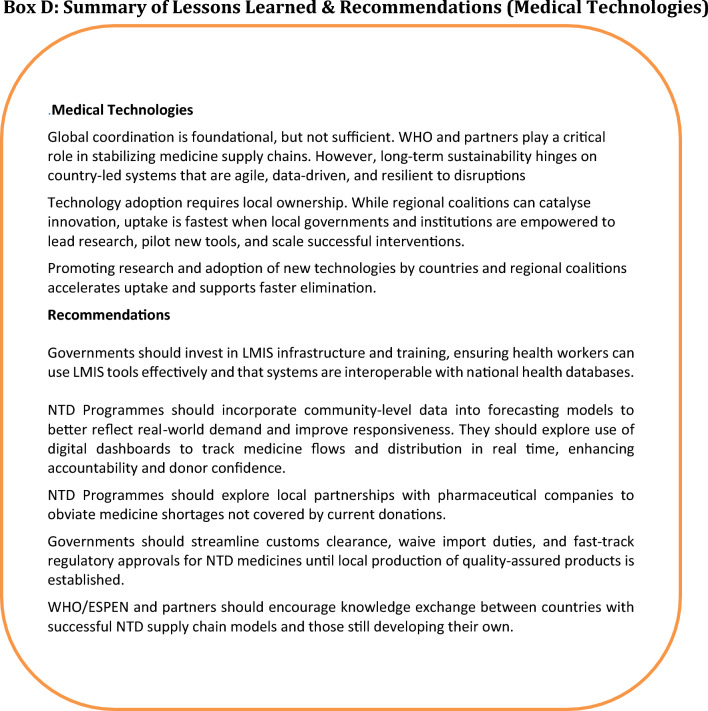
Summary of lessons learned and recommendations. Lessons learned and recommendations in the six health system building blocks are represented by: Box A: summary of lessons learned and recommendations (governance and healthcare financing), Box B: summary of lessons learned and recommendations (service delivery). Box C: summary of lessons learned (research and health workforce). Box D: summary of lessons learned and recommendations (medical products and new technologies)

The texts in Boxes A, B, C, and D (Fig. 4) outline key lessons learned and recommendations for each health system building block. Although some of the lessons learned were from programmes that existed years ago, they remain relevant, despite changing dynamics, in addressing the challenges that face NTDs’ elimination at the policy, planning, implementation, and surveillance stages.

Countries differ significantly in terms of achievements and challenges regarding infrastructure, human resources, governance, leadership, healthcare financing, technical capacity, service delivery, surveillance, technologies, and research. Therefore, countries and partners must conduct a thorough analysis of their specific situations and contexts, to enable them to adapt and prioritise actions and strategies that best meet their local NTDs’ elimination needs [133].

## Conclusions

Since its inception in 2016, the Expanded Special Project for the Elimination of Neglected Tropical Diseases (ESPEN) has played a pivotal role in advancing NTD control and elimination across Africa. Several countries have achieved interruption of transmission and halted mass drug administration (MDA) in all or selected endemic districts, with others attaining full geographical treatment coverage for preventive chemotherapy NTDs (PC-NTDs) [39, 134]. These gains reflect declining endemicity levels and demonstrate the effectiveness of regional coordination, drug donation mechanisms, and country ownership in accelerating progress toward the WHO 2021–2030 NTD Roadmap targets [1, 118], although progress remains uneven. Critical gaps persist in countries where MDA has not yet commenced or coverage remains insufficient, as seen in Gabon, where *Loa loa* co-endemicity poses a risk for adverse events associated with ivermectin administration [52]. In 2023, only 38% of individuals requiring treatment for schistosomiasis and around 40% for soil-transmitted helminthiases (STH) received treatment in Africa, while the continent accounted for 68.9% of the global population requiring onchocerciasis treatment [39, 50]. Similarly, 59% of individuals requiring MDA for lymphatic filariasis (LF) were treated, indicating significant programmatic gaps in countries such as Angola, the Central African Republic, and Nigeria [19].

These challenges are compounded by systemic health system weaknesses, including inadequate domestic financing, weak governance and partner coordination, fragile supply chain systems, and insufficient integration of NTD services into national health programmes. Drawing on lessons from historic successes such as the Onchocerciasis Control Programme (OCP), African Programme for Onchocerciasis Control (APOC), the Guinea Worm Eradication Programme, and the Global Polio Programme [68], strengthened leadership, strategic financing, resilient service delivery models, and effective community engagement are critical for sustaining gains and achieving elimination.

ESPEN’s experience underscores that NTD elimination is not solely a technical exercise but a health systems imperative. Strengthening the six WHO building blocks of leadership and governance, financing, service delivery, health workforce, information systems, and access to medicines and technologies is critical to driving sustained progress. By institutionalising NTD interventions within primary health care, enhancing cross-sectoral collaboration, and promoting country ownership, ESPEN and its partners are well situated to meet the 2030 Roadmap targets and contribute to universal health coverage and sustainable health systems across Africa.

## Data Availability

No datasets were generated or analysed during the current study.

## Abbreviations

ADAPT: Access, Determine, Add, Prioritise and Take-Stock
ALMA: African Leaders Malaria Alliance
APOC: African Programme for Onchocerciasis Control
CDC: Center for Disease Control and Prevention
CDDs: Community Drug Distributors
CDTI: Community directed treatment with ivermectin
CM: Case management
COVID-19: Coronavirus disease 2019
CSA: Committee of Sponsoring Agencies
DDT: Dichloro-diphenyl-trichloroethane
DEC: Diethyl carbamazine
DFRRI: Directorate of Food, Roads, and Rural Infrastructure
DIPs: District Implementation Plans
DMDi: Disease Management Disability & Inclusion
DNA: Deoxyribonucleic acid
DRC: Democratic Republic of Congo
EAC: Expert Advisory Committee
ESPEN: Expanded Special Project for the Elimination of Neglected Tropical Diseases
EPI: Expanded Programme on Immunisation
EVD: Ebola virus disease
GONE: Global Network for Onchocerciasis Elimination
GPEI: Global Polio Eradication Initiative
GIS: Geographical Information System
HCW: Health care worker
HIV: Human immunodeficiency virus
IDA: Ivermectin, DEC, and albendazole
IPTsc.: Intermittent Preventive Treatment for school-age children
iTAS: Integrated Transmission Assessment Survey
ITI: International Trachoma Initiative
IU: Implementation Unit
JAF: Joint Action Forum
JICA: Japan International Cooperation Agency
LF: Lymphatic filariasis
LMIS: Logistic Management Information System
MDA: Mass drug administration
M&E: Monitoring and evaluation
MERLA: Monitoring, Evaluation, Research, Learning and Adaptation
MMDP: Morbidity management and disability prevention
MMM: Mwele Malecela Mentorship
NGDO: Non-Governmental Development Organization
NGO: Non-Governmental Organisation
NIGEP: Nigeria Guinea Worm Eradication Programme
NOEC: National Onchocerciasis Elimination Committee
NOTF: National Onchocerciasis Task Force
NTDs: Neglected tropical diseases
OCP: Onchocerciasis Control Programme
OEM: Onchocerciasis Elimination Mapping
OEPA: Onchocerciasis Elimination Programme for the Americas
PC: Preventive chemotherapy
PC-NTDs: Preventive chemotherapy for neglected tropical diseases
PCR: Polymerase chain reaction
PHC: Primary health care
PMC: Perennial malaria chemoprevention
RAPLOA: Rapid assessment procedure for loiasis
REMO: Rapid Epidemiological Mapping of Onchocerciasis
RLMF: Reaching the Last Mile Fund
RPAG: Regional Programme Advisory Group
SAFE: Surgery, antibiotics, facial cleanliness and environmental cleanliness
SBCC: Social behaviour change communication
SDGs: Sustainable Development Goals
SIZ: Special Intervention Zone
SMC: Seasonal malaria chemoprevention
STH: Soil-transmitted helminths
TAS: Transmission assessment survey
TB: Tuberculosis
TCC: Technical Consultative Committee
TDR: Special Programme for Research and Training in Tropical Diseases
UNICEF: United Nations Children’s Fund
US: United States
USAID: United States Agency for International Development
WASH: Water, sanitation and hygiene
WHO: World Health Organization
